# Baicalin Inhibits Influenza A Virus Infection *via* Promotion of M1 Macrophage Polarization

**DOI:** 10.3389/fphar.2020.01298

**Published:** 2020-10-06

**Authors:** Ping Geng, Haiyan Zhu, Wei Zhou, Chang Su, Mingcang Chen, Chenggang Huang, Chengjie Xia, Hai Huang, Yiou Cao, Xunlong Shi

**Affiliations:** ^1^Department of Biological Medicines & Shanghai Engineering Research Center of Immunotherapeutics, Fudan University School of Pharmacy, Shanghai, China; ^2^Department of Chemistry, Fudan University, Shanghai, China; ^3^Department of Surgery, Minhang Hospital, Fudan University, Shanghai, China; ^4^Shanghai Institutes of Materia Medica, Chinese Academy of Sciences, Shanghai, China

**Keywords:** macrophage, M1 polarization, influenza virus, baicalin, metabolism

## Abstract

**Background and Aims:**

The natural compound baicalin (BA) possesses potent antiviral properties against the influenza virus. However, the underlying molecular mechanisms of this antiviral activity and whether macrophages are involved remain unclear. In this study, we, therefore, investigated the effect of BA on macrophages.

**Methods:**

We studied macrophage recruitment, functional phenotypes (M1/M2), and the cellular metabolism *via* flow cytometry, qRT-PCR, immunofluorescence, a cell culture transwell system, and GC-MS–based metabolomics both *in vivo* in H1N1 A virus-infected mice and *in vitro*.

**Results:**

BA treatment drastically reduced macrophage recruitment (CD11b^+^, F4/80^+^) by approximately 90% while maintaining the proportion of M1-polarized macrophages in the bronchoalveolar lavage fluid of infected mice. This BA-stimulated macrophage M1 phenotype shift was further verified *in vitro* in ANA-1 and primary peritoneal macrophages by measuring macrophage M1 polarization signals (CD86, iNOS, TNF-α, *iNOS/Arg-1* ratio, and IL-1β cleavage). Meanwhile, we observed an activation of the IFN pathway (upregulation of *IFN-β* and *IRF-3*), an inhibition of influenza virus replication (as measured by the *M* gene), and distinct cellular metabolic responses in BA-treated cells.

**Conclusion:**

BA triggered macrophage M1 polarization, IFN activation, and other cellular reactions, which are beneficial for inhibition of H1N1 A virus infection.

## Introduction

Influenza viruses are the most frequent human pathogens and are responsible for many complications, such as viral pneumonia (Rello, 2017; http://www.who.int/influenza/surveillance_monitoring/updates/latest_update_GIP_surveillance/en/). Novel mutant influenza A viruses, such as avian H7N9, do not respond well to current antiviral therapies and can cause substantial excess mortality during pandemics and the annual flu season (Zhou et al., 2017). Thus, the development of novel therapeutic strategies is an urgent matter.

Macrophages are one of the primary sources of inflammatory cytokines (such as TNF-α/chemokines) and act as critical modulators of influenza virus infection severity and the development of lethal pulmonary injury (Cardani et al., 2017). Their phenotype—classically activated (M1) or alternatively activated (M2)—is recognized as a key regulating factor in the initiation, progression, and termination of numerous inflammatory diseases (Li et al., 2018), in particular in influenza diseases (Arora et al., 2018). Therefore, modulating macrophage polarization might represent a possible therapeutic strategy for influenza virus infection (Vergadi et al., 2017; Halstead et al., 2018).

Chemicals with anti-influenza activity derived from natural products are rapidly emerging, some of which may constitute possible novel drug options (Cardani et al., 2017). Baicalin (BA) is a natural flavonoid present in several medicinal plants and is shown to prevent death, inhibit lung consolidation, and reduce viral replication (Ding et al., 2014; Nayak et al., 2014; da Silva et al., 2017). In a previous study, we found that BA could inhibit influenza virus–induced autophagy in macrophages (Zhu et al., 2015). However, it is still unknown whether BA exerts its antiviral effects *via* modulation of macrophage functions.

Reprogramming of the host cellular metabolism triggered by the influenza virus is highly associated with inflammation and immune cell activation (Chandler et al., 2016), and metabolomics is believed to be a highly sensitive and specific tool for the prognosis of viral pneumonia in anti-influenza clinical therapies (Tisoncik-Go et al., 2016). In this research, we use metabolomics (GC-MS analysis) to evaluate the effects of BA on macrophages.

## Materials and Methods

### Chemicals and Reagents

BA (98% HPLC purity; ChromaBio, Chengdu, China) was dissolved in DMSO to generate a 50-mM stock solution and stored at 4°C. It was subsequently diluted in cell culture medium to different working solutions. For animal experiments, BA and ribavirin solutions were prepared with 0.5% CMC-Na.

Antibodies against mouse CD11b, F4/80, CD86, CD206, and cleaved interleukin-1β (IL-1β) were obtained from Biolegend (San Diego, CA, USA). Secondary antirabbit IgG Fab2 Alexa Fluor ^®^ 488 molecular probe was purchased from Santa Cruz Biotechnology (Santa Cruz, CA, USA). All other chemicals were obtained from Beyotime Biotechnology (Shanghai, China).

## Methods

### Experimental H1N1 Infection

Male BALB/c mice (16–18 g) were purchased from the Shanghai SLACCAS Laboratory Animal Co., Ltd. (Shanghai, China). Mice were housed under specific pathogen-free (SPF) conditions and given free access to sterile water and standard mouse chow. All experimental protocols were approved by the Animal Experiment Committee of Fudan University (Shanghai, China) (Approval number 2018-03-WY-SXL-01).

The influenza virus A/FM/1/47 (H1N1) was supplied by the Shanghai Center for Disease Control & Prevention (Shanghai, China) and was stored in aliquots at -70°C. For each experiment, an aliquot was thawed to ensure the use of a fresh preparation.

Mice were randomly divided into four groups with 8 mice per group: control mice (Normal group), virus-infected mice (Virus group), BA-treated infected mice (BA group), and ribavirin-treated infected mice (Ribavirin group). All mice except control mice were anesthetized under 1% pentobarbital and infected intranasally (i.n.) with 5×LD_50_ of A/FM/1/47 (H1N1). Two hours postinfection, mice were treated with 80 mg/kg of BA (BA group) (Ding et al., 2014), 20 mg/kg of ribavirin (Ribavirin group) (Ridnour et al., 2015), and 0.5% CMC-Na (Normal and Virus groups) by gavage once daily for 7 days. The experiments were repeated twice, and all mice were continuously monitored for survival and body weight loss.

### Experimental H1N1 Infection in Macrophages and Epithelial Cells

For *in vitro* experiments, the H1N1/PR/8/34 TC adapted strain was obtained from ATCC (VR,1469 AC) and stored in aliquots at -70°C. For each experiment, an aliquot was thawed to ensure the use of a fresh preparation.

Murine ANA-1 macrophages and epithelial BEAS-2B cells were obtained from the Cell Bank of the Shanghai Institute of Biochemistry and Cell Biology of the Chinese Academy of Sciences (Shanghai, China).

Murine ANA-1 macrophages (Fabozzi et al., 2018) and primary peritoneal macrophages (isolated from SPF Balb/C mice) were cultured in RPMI 1640 medium (1×10^5^ cells/ml) with 10% (v/v) heat-inactivated fetal bovine serum (Gibco), 0.3 mg/mL l-glutamine, 100 U/mL penicillin, and 100 μg/mL streptomycin at 37°C under 5% CO_2_.

BEAS-2B cells (Weinhofer et al., 2018) were cultured in the DMEM supplemented with 10% (v/v) heat-inactivated fetal bovine serum (Gibco), 0.3 mg/mL l-glutamine, 100 U/mL penicillin, and 100 μg/mL streptomycin at 37°C under 5% CO_2_.

The cells were infected with 10× the median tissue culture infectious dose (10× TCID_50_) of H1N1 virus (PR/8/34, ATCC) for 2 h and then transferred to BA solution (diluted in 1640 medium for macrophages and DMEM for BEAS-2B cells) for 24 or 48 h.

### The Phenotype of Macrophages In Vivo and In Vitro

For the *in vivo* study, the bronchoalveolar lavage fluid (BALF) of different grouped mice was collected three times by lavage with 0.5 mL ice-cold PBS under pentobarbital anesthesia at 4 days postinfection. The collected BALF was centrifuged at 700×*g* at 4°C for 5 min, and the harvested cells were resuspended in 200 μL PBS. The cells were stained with fluorescently labeled antibodies against the following mouse proteins: CD11b^+^, F480^+^ (macrophages), CD11b^+^, F4/80^+^, CD86^+^ (M1 phenotype macrophage), and CD11b^+^, F4/80^+^, CD206^+^ (M2 phenotype macrophage) (Schmittgen and Livak, 2008; Murray et al., 2014).

For the *in vitro* study (24 and 48 h postinfection), infected ANA-1 macrophages were stained with fluorescently labeled antibodies against the following mouse proteins: CD86^+^ (M1 phenotype) and CD206^+^ (M2 phenotype).

Flow cytometry analysis was performed on a BD FACS Calibur (BD Biosciences, USA). All plots shown on populations were gated on lymphocytes by forward and side scattering, collecting 100,000 cells per sample. Compensation settings were adjusted using isotype controls. Data analysis was performed using the FlowJo software (Ashland, OR, USA).

High levels of cellular iNOS and TNF-α are often observed in M1 polarized macrophages. Thus, we quantified cellular iNOS (using the Diaminofluorescein-FM diacetate probe; Nitric Oxide Synthase Assay Kit, S0025, Beyotime, Shanghai, China) and TNF-α (using a TNF-α ELISA kit) to evaluate the macrophage polarization.

### Real-Time Quantitative RT-PCR (qRT-PCR) Analysis

For cell experiments at 24 and 48 h postinfection, total RNA was isolated using TRIzol reagent (Invitrogen, CA, USA) according to the manufacturer’s instructions. The RNA was converted into cDNA using a Reverse Transcription Kit (Takara, Japan). qRT-PCR was performed using the StepOne Plus RT-PCR System (Applied Biosystems) using the following thermocycling parameters: 94°C for 5 min, followed by 40 cycles of 94°C for 5 s and 60°C for 30 s. The mRNA levels of *iNOS*, *Arg-1*, *influenza M* gene, *INF-β*, *IL-10*, *IRF-3*, and *IRF-7* were normalized to the geometric mean of *GAPDH* mRNA levels by using a ΔΔCT method (Lin et al., 2010). The primers used in this study are shown in **Table 1**.

**Table 1:** Primers for qPCR.

Primer name	Sequence (5’to3’)	
Influenza VirusM gene	Forward	GACCGATCCTGTCACCTCTGAC
	Reverse	AGGGCAT CTGGACAAAGCGTCTA
iNOS	Forward	TCCTGGAGGAAGTGGGCCGAAG
	Reverse	CCTCCACGGGCCCGGTACTC
Arg-1	Forward	CAGAAGAATGGAAGAGTCAG
	Reverse	CAGATATGCAGGGAGTCAC
IRF3	Forward	TCCGCTTAGTCTACAGCCCT
	Reverse	ACACTGTTACCTGATCTGCCC
IRF7	Forward	CAGCTCAGCAGCCTTACCAC
	Reverse	TGACATTGGCGCTGTGAAGAG
IL-1β	Forward	TGCCACCTTTTGACAGTGATG
	Reverse	TGATGTGCTGCTGCGAGATT
IL-10	Forward	GGACAACATACTGCTAACCGAC
	Reverse	TGGATCATTTCCGATAAGGCTTG
IFN-β	Forward	TGCATCTTCTCCGTCATCTC
	Reverse	TAGCAGCCGACACCAGCCTG
GAPDH	Forward	ACAGCCTCAAGATCATCAGCA
	Reverse	ATGAGTCCTTCCACGATACCA

### Macrophage Recruitment Assay (Transwell System)

ANA-1 macrophages (labeled with GFP lentiviral vectors from Genechem, Shanghai, China) were cultivated in the upper chamber of 24-well Transwell plates (10^4^ cells/well). ANA-1 or BEAS-2B cells were grown in the lower room (10^5^ cells/mL). The cells in lower chambers were infected with the influenza PR/8/34 virus (10×TCID_50_) for 2 h and then transferred to fresh culture medium or BA dilutions.

Macrophage recruitment was observed under a Leica EL6000 microscope (Leica Microsystems CMS GmbH).

### Cell Immune-Fluorescence Staining Analysis

Cells were fixed with 4% paraformaldehyde buffer for 30 min, permeabilized with 0.5% Triton X-100 for 20 min, and blocked with QuickBlock™ Blocking Buffer for 30 min at room temperature. The cells were probed with IL-1β antibodies (1:1000 dilution) at 4°C overnight and subsequently detected with antirabbit IgG Fab2 Alexa Fluor ^®^ 488 molecular probes (1:2000 dilution). After counterstaining cell nuclei with DAPI (1:1000 dilution of 100 µg/mL stock solution) for at least 3 min, immunofluorescent images were obtained using a Leica EL6000 microscope (Leica Microsystems).

### Metabolomics

The cell samples were frozen and thawed at room temperature. Three hundred μL methanol were added to 100 μL of the homogenate to precipitate the protein, and the mixture was shaken vigorously for 30 s. Ribitol (10 µL at 0.02 mg/mL) was added to the sample as the internal standard. The mixture was then ultrasonically extracted for 10 min, followed by centrifugation (3000 × g) for another 10 min. Then, 150 μL of the supernatant was transferred to the GC vial and evaporated to dryness under a stream of nitrogen gas. The chemical derivatization of the cell metabolites was carried out using the combination of methoxymation and silylation. A 30-μL aliquot of methoxyamine pyridine solution (20 mg/mL) was added to the vial. The methoxymation was performed at 37°C for 90 min. Then, 30 μL of bis-(trimethylsilyl)–trifluoroacetamide plus 1% trimethylchlorosilane were added to each sample. The samples were mixed on a vortex for 1 min and incubated at 70°C for 60 min. After incubation, samples were again vortexed for 1 min and transferred to vials for GC/MS analysis (Agilent 7890A GC system).

The GC/MSD Chem Station Software (Agilent, Shanghai, China) was used for the autoacquisition of GC total ion chromatograms and fragmentation patterns. For each peak, the software generates a list of similarities comparing with every substance within the National Institute of Standards and Technology (NIST) mass spectra library. Peaks with a similarity index of more than 70% were assigned compound names, and those having less than 70% similarity were listed as unknown metabolites. Each sample was characterized by the same number of variables, and each of these variables was represented across all observations with the same sequence. Thus, a data matrix was generated by the intensities of the commensal peaks from all samples to characterize the biochemical pattern of each sample (Wang et al., 2016).

To create the metabolite heat map, we normalized data for each metabolite [(original value-mean)/standard deviation], obtaining a *z*-score, and then set the minimum value to -2 (dark blue), the median to 0 (white), and maximum value to 2 (dark red).

### Statistical Analysis

All statistical analyses were performed using GraphPad Prism for Windows (Version 6.0). The Gehan-Breslow-Wilcoxon test was used to analyze the survival of mice, and other experimental data were evaluated by using the Student’s *t*-test (comparison of two groups) or one-way ANOVA (for comparisons with more than two groups sample). In all cases, probability levels less than 0.05 (*P* < 0.05) were considered to indicate statistical significance.

## Results

### BA Significantly Inhibits the Recruitment of Macrophages Into the Lung Tissues of H1N1-Infected Mice

**Figure 1:**
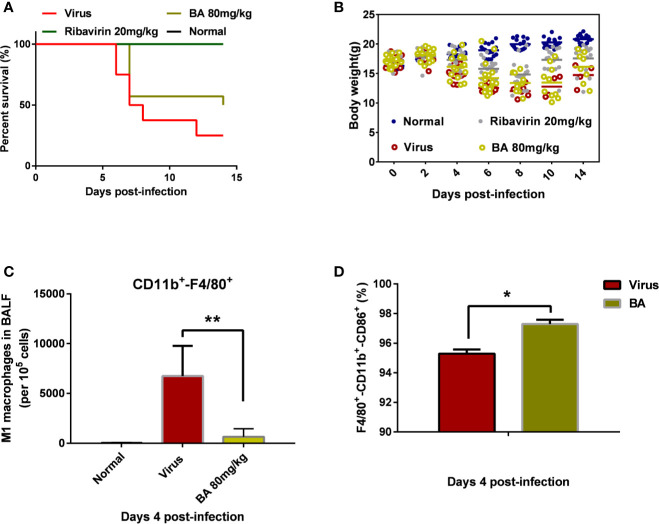
BA reduces the mortality of infected mice and suppresses the recruitment of macrophages in BALF. BALB/c mice were i.n. infected with influenza virus A H1N1 FM/1/47 strain at 5×LD_50_ and received BA treatment (80 mg/kg/day) for 7 days. **(A)** Survival curve. **(B)** Body weight curve. **(C–D)** Macrophage phenotype in the BALF (Normal, Virus, and BA treatment, 4 days postinfection). Data are presented as mean ± SD. *p ≤ 0.05, **p ≤ 0.01 compared with virus samples.

As shown in **Figure 1A**, the infection with 5×LD_50_ influenza virus A/FM/1/47 caused 75% mortality within 14 days postinfection, accompanied by visible body weight loss. Treatment with 80 mg/kg BA attenuated death and body weight loss of infected mice (**Figure 1B**).

We collected the BALF of infected mice (control, virus, BA-treated) at 4 days postinfection and analyzed macrophage recruitment and phenotype. As shown in **Figure 1C**, very few macrophages were present in lung tissues of normal mice although H1N1 infection caused the massive recruitment of macrophages (nearly 10% CD11b^+^-F4/80^+^ macrophages per 10^5^ cells) to the lungs. BA treatment (80 mg/kg) significantly reduced virus-induced macrophage recruitment (1% of 10^5^ cells). Furthermore, we then analyzed the polarization of recruited macrophages from the virus and BA groups and found that BA treatment resulted in a higher proportion of M1 macrophages than in virus mice (97% CD11b^+^-F4/80^+^-CD86^+^ triple positive vs. 95% triple positive) as shown in **Figure 1D**.

We, therefore, concluded that recruitment and polarization of macrophages might play a critical role in the antiviral effects of BA.

### BA triggers macrophage M1 polarization and activates IFN signaling pathway

**Figure 2:**
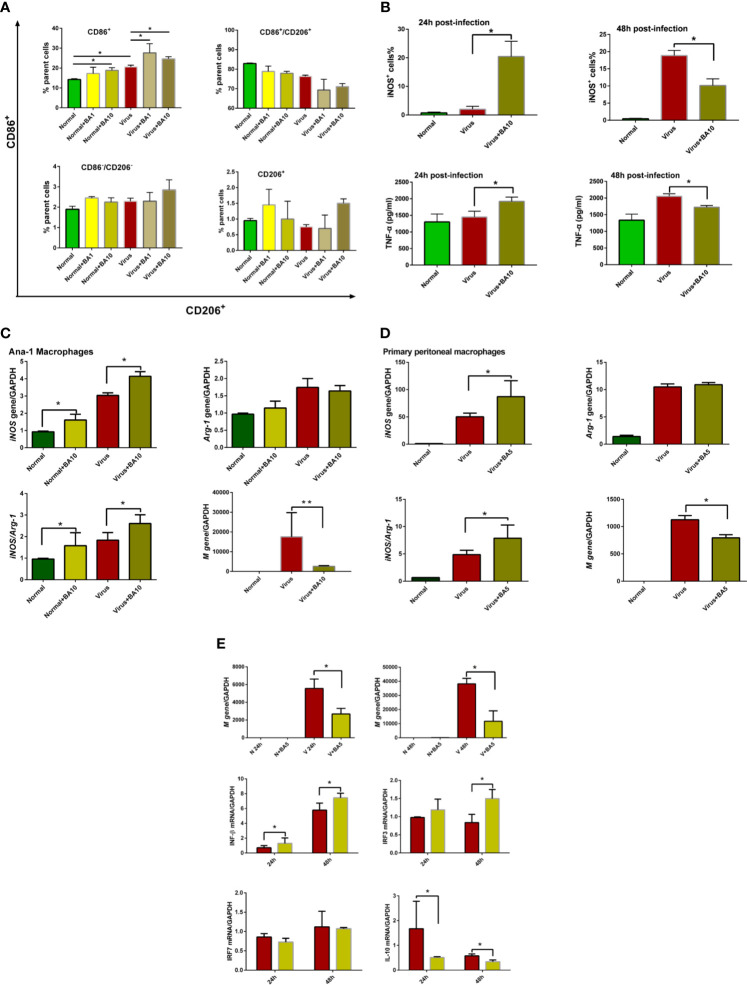
triggers macrophage M1 polarization and activates the IFN signaling pathway. Macrophages (ANA-1 cells and primary peritoneal macrophages, 2×10^5^ cells/well) were infected with influenza virus H1N1 PR/8/34 strain (10×TCID_50_) and cultured in 6-well plates. The medium was replaced by BA dilutions (1 and 10 μM) after 2 h of incubation. Uninfected cells were used as the standard control. **(A)** Macrophage polarization of ANA-1 cells. At 24 h postinfection, ANA-1 cells were stained with fluorescently labeled antibodies against the following mouse proteins: CD86^+^ (M1 phenotype) and CD206^+^ (M2 phenotype). The population of labeled cells was analyzed by flow cytometry. **(B)** At 24 and 48 h postinfection, cellular iNOS and secreted TNF-α were further quantified by Nitric Oxide Synthase Assay Kit and ELISA Kit. **(C, D)** At 24 h postinfection, total RNA of cell samples (10 μM BA treated-ANA-1 cells; 5 μM BA treated-primary peritoneal macrophages) was isolated and reverse-transcribed to cDNA for analysis of macrophage phenotype (*iNOS* and *Arg-1* gene) and influenza virus replication (*M* gene). **(E)** Comparative analysis of IFN, IRFs, and other related gene transcription in ANA-1 cell samples (24 and 48 h postinfection). Data are presented as mean ± SD. *p ≤ 0.05, **p ≤ 0.01 compared with virus samples.

ANA-1 macrophages are a commonly used murine macrophage cell line (Evavold et al., 2018), which we previously used to replicate influenza virus infection *in vitro* (Zhu et al., 2015). Therefore, we used ANA-1 cells to evaluate macrophage recruitment and polarization after H1N1 PR/8/34 infection (10 TCID_50_) with or without BA treatment. As shown in **Figure 2A**, regardless of whether cells were healthy or infected, BA treatment (1 and 10 µM) promoted an M1 polarization of macrophages by enhancing CD86 positive cells, but had a limited effect on other macrophages (such as CD86-CD206 double-positive, CD86-CD206 double-negative, and CD206 single positive macrophages).

In **Figure 2B**, we show that BA treatment (10 µM) significantly enhanced cellular iNOS and TNF-α levels in infected ANA-1 cells at 24 h postinfection but decreased iNOS levels at 48 h postinfection.

We further analyzed macrophage M1 phenotype-related gene expression (*iNOS* and *Arg-1*) in ANA-1 macrophages and primary peritoneal macrophages. In ANA-1 cells, BA (10 µM) markedly upregulated the *iNOS* transcription, increased the *iNOS/Arg-1* ratio, and suppressed influenza *M* gene replication at 24 h postinfection (**Figure 2C**). A lower dose of BA (5 µM) also showed similar effects in primary peritoneal macrophages (**Figure 2D**).

We further evaluated the effects of BA (5 µM) on the IFN signaling pathway in ANA-1 cells at 24 and 48 h postinfection. Influenza virus replicated in ANA-1 cells in a time-dependent manner, which was inhibited by BA treatment. We further compared *IFN-β*, *IL-10*, and interferon regulatory factor 3/7 (*IRF-3/7*) between untreated infected and BA-treated infected cells. The results indicate that BA treatment upregulates *IFN-β* and *IRF-3* transcription and downregulates transcription of *IL-10* as shown in **Figure 2E**.

These results suggest that BA exerts its suppression of viral replication at an early stage by triggering macrophage M1 polarization and activating IFN signaling.

### BA Promotes IL-1β Cleavage and Cell–Cell Adhesion

The IL-1 cytokine family cytokines are cytosolic proteins that exhibit inflammatory activity upon release into the extracellular space. The mature, cleaved form of IL-1β is closely related to the hyperactivated macrophage (Fabozzi et al., 2018).

**Figure 3:**
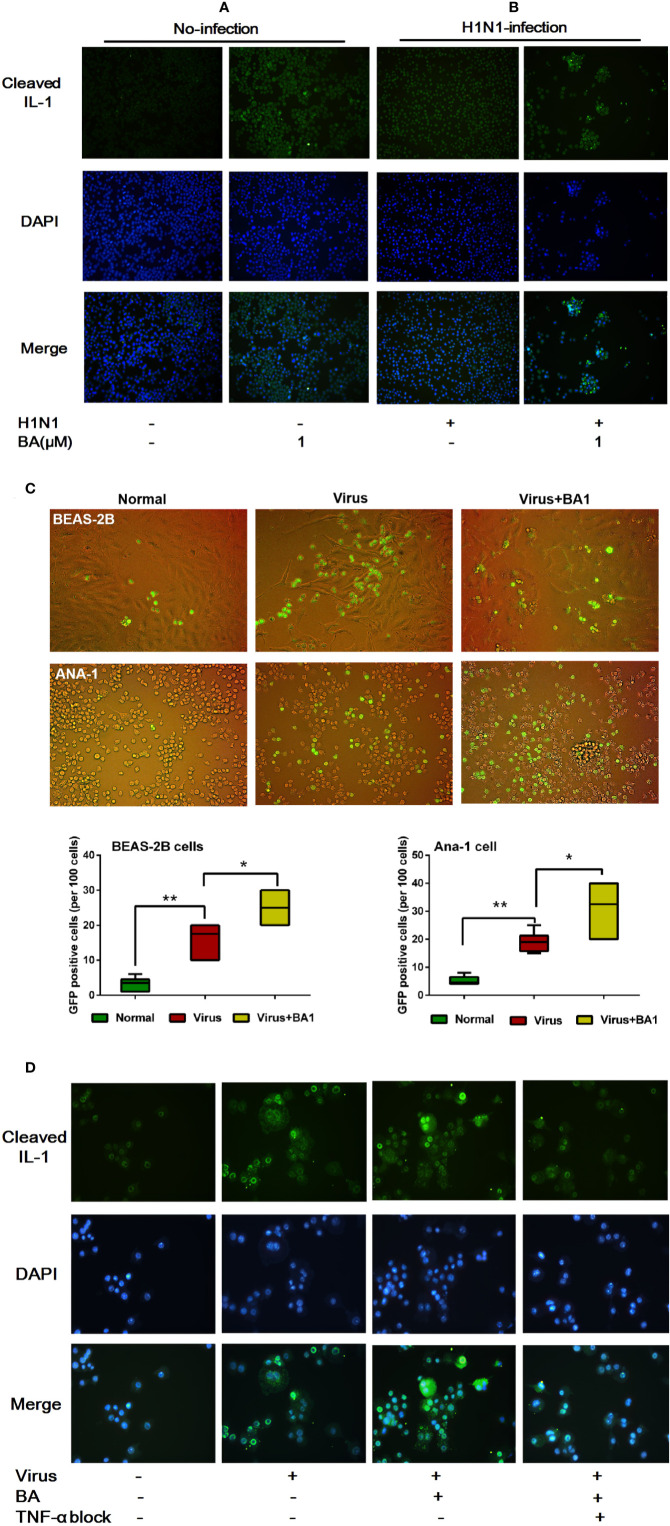
BA amplifies influenza-induced macrophage IL-1β cleavage and cell–cell adhesion. Cells were infected with influenza virus H1N1 PR/8/34 strain at 10×TCID50. The medium was replaced after 1.5 h incubation, and then BA dilutions were added to 6-well plates. Uninfected cells were used as the standard control. **(A, B)** At 24 h postinfection, ANA-1 cells were stained with a fluorescently labeled antibody against cleaved IL-1β. The cell nucleus was counterstained with DAPI. **(C)** In the Transwell system, GFP-labeled ANA-1 cells were inoculated in the upper chambers; the bronchial epithelial cells (BEAS-2B), and macrophage cells (ANA-1) in the lower chamber were infected and treated with BA. Macrophage recruitment and cell–cell adhesion were imaged using a Leica EL6000 microscope (Leica Microsystems CMS GmbH). **(D)** Infected ANA-1 cells were treated with BA and Etanercept (TNF-α inhibitor). IL-1β cleavage and cell–cell adhesion were observed under a Leica EL6000 microscope (Leica Microsystems CMS GmbH). Data are presented as mean ± SD. *p ≤ 0.05, **p ≤ 0.01 compared with virus samples.

We assessed the activation of IL-1β in ANA-1 cells at 24 h postinfection by immunofluorescence. As shown in **Figure 3A**, we observed that 1 µM BA could increase levels of cleaved IL-1β. In infected cells, BA significantly promoted virus-induced IL-1β cleavage (**Figure 3B**). Meanwhile, BA also significantly enhanced cell–cell adhesion to form macrophage aggregates.

To explore the influence of BA on macrophage migration, we used wild-type and GFP^+^ ANA-1 macrophages and human BEAS-2B cells (Cardani et al., 2017) in the Transwell experimental system (8.0 µm membrane). By tracking GFP^+^ ANA-1 cell migration, we observed that H1N1 infection resulted in an enhanced GFP^+^ ANA-1 cell recruitment to infected BEAS-2B and macrophages (**Figure 3C**). BA treatment improved GFP^+^ cell aggregation, and we detected more damaged cells (abnormal morphology) in infected BEAS-2B and ANA-1 cells.

To evaluate the role of TNF-α in BA-treated macrophages, we then used Etanercept, which is a commercially available soluble TNFR fusion protein inhibiting TNF-α at 70 µg/mL. TNF-α inhibition appreciably abolished IL-1β cleavage and cell–cell adhesion triggered by BA (**Figure 3D**).

This evidence suggested that BA potentiated the hyperactivation and cell–cell adhesion of infected macrophages, and our results indicate that IL-1β and TNF-α possibly played a crucial role in this process.

### BA Triggers Discriminative Cellular Metabolic Responses

The metabolic response plays a central role in linking cell responses in influenza virus–infected cells. Here, we used GC-MS–based metabolomics to analyze the effects of BA treatment on the metabolic profile of ANA-1 cells.

**Figure 4:**
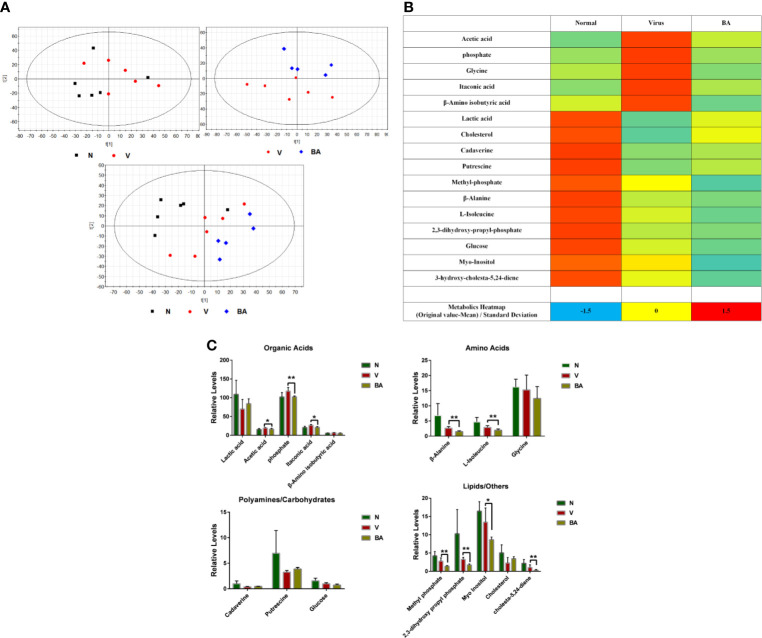
Metabolomics analysis. At 48 h postinfection, cell samples were harvested for metabolomic analysis. **(A)** Partial least squares discriminant analysis (PLS-DA) was used to identify the obtained 1977 metabolite markers from GC-MS (N: healthy cells; V: virus-infected cells; BA: the infected cells treated by BA). **(B)** Metabolic heat map. For each metabolite, data was normalized using the *z*-score ((original value-mean) standard deviation) to values between -2 (dark blue), 0 (median; white), and 2 (maximum, dark red), to make a metabolite heat map. **(C)** Comparative analysis of major metabolites. The 16 metabolites were grouped as organic acids, amino acids, polyamines/carbohydrates, lipids/others. Data are presented as mean ± SD. *p ≤ 0.05, **p ≤ 0.01 compared with virus samples.

Cell samples were harvested for metabolomics analysis at 48 h postinfection. We obtained 1977 metabolite markers from GC-MS by peak selection and alignment against the NIST library (only peaks with more than 70% similarity were considered as “detected”). Partial least squares discriminant analysis (PLS-DA) was undertaken to identify the differential metabolites of different groups (N: normal cells, V: virus-infected cells, BA: infected cells treated with BA). BA treatment resulted in a noticeable deviation of metabolic responses in infected ANA-1 cells compared with normal and virus-infected samples (**Figure 4A**).

We identified 25 endogenous metabolites between normal and virus control samples, 17 endogenous metabolites between virus and BA samples, and explored the overall deviation in metabolism *via* a metabolite heat map. Compared to normal samples, the level of most metabolites in the virus-infected samples visibly increased, indicating that viral infection accelerated most metabolic reactions, and BA triggered a deviation of metabolites in infected samples (**Figure 4B**).

We identified 16 endogenous metabolites, which were significantly different between normal, virus control, and BA-treated cells. These metabolites covered, among others, organic acids, carbohydrates, amino acids, and lipids, which indicated that virus infection disturbed the overall metabolism. We, therefore, grouped metabolites as organic acids, amino acids, polyamines/carbohydrates, and lipids/others as shown in **Figure 4C**. In the group of organic acids, BA reversed the upregulated levels of acetic acid, phosphate, and itaconic acid in infected samples. Apart from this, BA accelerated the degradation of amino acids (β-alanine, L-isoleucine) and lipids/others (methyl phosphate, 2,3-dihydroxy propyl phosphate, cholesta-5,24-diene) in infected samples.

Thus, BA resulted in a complicated metabolic response in ANA-1 cells at 48 h postinfection, which was possibly linked to its immune-stimulating effects on macrophages.

## Discussion and Conclusion

Using both *in vitro* and *in vivo* models, we demonstrate in this study that BA treatment could ameliorate H1N1 A virus–induced mortality *via* promotion of macrophage M1 polarization and IFN signaling pathway activation in the early stages of infection.

Macrophages are critical modulators of influenza A virus disease severity and the subsequent development of lethal pulmonary injury (Arora et al., 2018). Their shift between the classically activated (M1, proinflammatory) and alternatively activated (M2, anti-inflammatory) phenotypes has been recognized as a crucial factor in the initiation, progression, and termination of numerous inflammatory diseases (Zhang et al., 2018) and especially in influenza virus infection (Halstead et al., 2018). Modulation of macrophage polarization has previously been shown to inhibit or promote influenza infection by applying GM-CSF (Sun et al., 2018) or Afatoxin B1 (Han et al., 2019), respectively. In this study, we confirm that the high mortality of infected mice is correlated with an abundant recruitment of macrophages to the lung and a high proportion of M1-polarized macrophages. BA treatment alleviated body weight loss and death of infected mice, and significantly reduced the recruitment of macrophages to the lung as measured by flow cytometry on the BALF. Meanwhile, we observed that BA treatment resulted in a maintained high proportion of M1-polarized macrophages. To elucidate the underlying molecular mechanisms of BA action, we next performed several *in vitro* experiments.

In murine ANA-1 and primary peritoneal macrophage cells, we further verified the M1 activation by BA treatment in multiple experiments (flow cytometry for CD86/CD206 and iNOS, ELISA for TNF-α, and qPCR to determine the *iNOS/Arg-1* ratio). This M1 polarization by BA after infection echoed similar M1-promoting effects of BA in the tumor microenvironment (Li and Wang, 2019). At the same time, we found that BA treatment promoted the upregulation of *IFN*-*β* and *IRF3* transcripts in ANA-1 cells, which is in line with other findings demonstrating IFN signaling pathway activation by BA treatment (Chu et al., 2015; Di Paolo and Shayakhmetov, 2016). Thus, macrophage M1 polarization and IFN signaling activation might be a key to the antiviral effects of BA.

In this study, we also evaluated macrophage M1 polarization by assessing IL-1 cleavage, which corresponds to macrophage hyperactivation (Fabozzi et al., 2018) and is involved in numerous inflammatory diseases (Ilyas et al., 2016). Through cellular immunofluorescence, we found that BA remarkably amplified the cleavage of IL-1β and cell–cell adhesion of the infected cells, which further verified macrophage M1 polarization. In our previous work, we demonstrated that BA treatment resulted in inhibitory effects on autophagosome accumulation in infected macrophages. Macrophage autophagy could limit acute toxic liver injury in mice through downregulation of IL-1β (Wang A. et al., 2016). Autophagy might be involved in the promotion of macrophage M1 polarization by BA, but the underlying mechanisms need to be further clarified in future experiments.

Viral infection triggers a cellular metabolic reprogramming, which is linked to immunity (Chen et al., 2014; Smallwood et al., 2017). In our previous work, we successfully used metabolomics to evaluate the systematic influence of a multicomponent natural product preparation on influenza virus infection (Sithisarn et al., 2013). Here, we also used GC-MS–based metabolomics to explore the impact of baicalin on macrophages in influenza virus infection. The metabolomic data presented in metabolic heat maps and PLS-DA showed that BA induced significant differentiation of metabolites, deviating from normal and virus-infected samples. BA treatment mainly promoted a reduction of amino acids (β-alanine, L-isoleucine) and lipids/others (methyl phosphate, 2,3-dihydroxy propyl phosphate, cholesta-5,24-diene), consistent with BA promoting an enhanced macrophage M1 polarization. The metabolomics data in this study provide a systematic evaluation; we aim to further clarify how metabolism and specific metabolites link to macrophage M1 polarization in further experiments.

The antiviral and anti-inflammatory effects of flavonoids have been reported in many studies (Zhi et al., 2019; Kiruthiga et al., 2020), but the underlying mechanism of these properties remains unclear. Regulation of macrophage recruitment and polarization is a promising field of research, and it may be beneficial to promote the research and development of flavonoids for antiviral and anti-inflammatory therapeutic applications.

## Data Availability Statement

The datasets presented in this study can be found in online repositories. The names of the repository/repositories and accession number(s) can be found in the article/supplementary material.

## Ethics Statement

The animal study was reviewed and approved by The Animal Experiment Committee of Fudan University (Shanghai, China).

## Author Contributions

PG and HZ carried out all in vitro and in vivo experiments in this study. WZ and HH carried out the immunoassays. CS, MC and CX performed the data analysis. CH participated in the design of the study. YC and XS conceived the study, participated in its design and coordination and helped to draft the manuscript. All authors contributed to the article and approved the submitted version.

## Funding

This work was supported by the Key Program of Nation Nature Science Foundation of China (No.U1604283), Shanghai Science and Technology Funds (Nos. 17ZR1401700), and Shanghai Municipal Health Commission project (Nos.201740200).

## Conflict of Interest

The authors declare that the research was conducted in the absence of any commercial or financial relationships that could be construed as a potential conflict of interest.
