# Supplementary material for: Baicalin Inhibits Influenza A Virus Infection via Promotion of M1 Macrophage Polarization
Source: Front Pharmacol. 2020 Oct 6;11:01298. doi: 10.3389/fphar.2020.01298 (PMC7574031; doi:10.3389/fphar.2020.01298)
Supplement: Supplementary file 1 [file DataSheet_1.docx]

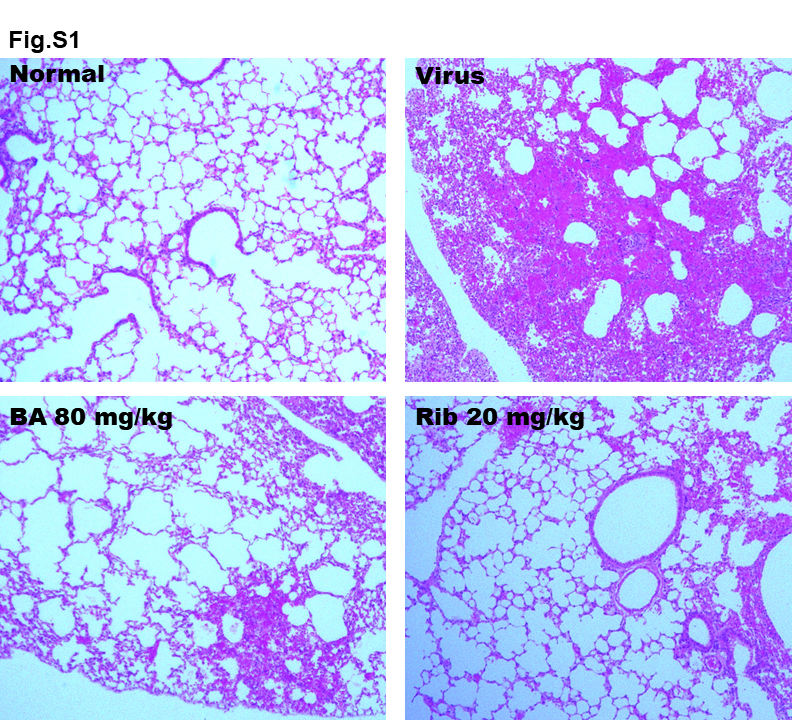


On day four post-infection, the mice (n = 6, per group) were euthanized. Lung tissues were harvested for histopathology. The left lobes of the lung were suspended in PBS-buffered formalin. They were then preserved in paraffin blocks using standard procedures. 10-µm tissue sections were cut, placed on glass slides, and stained with hematoxylin and eosin (HE) using conventional techniques.

Fig.S2


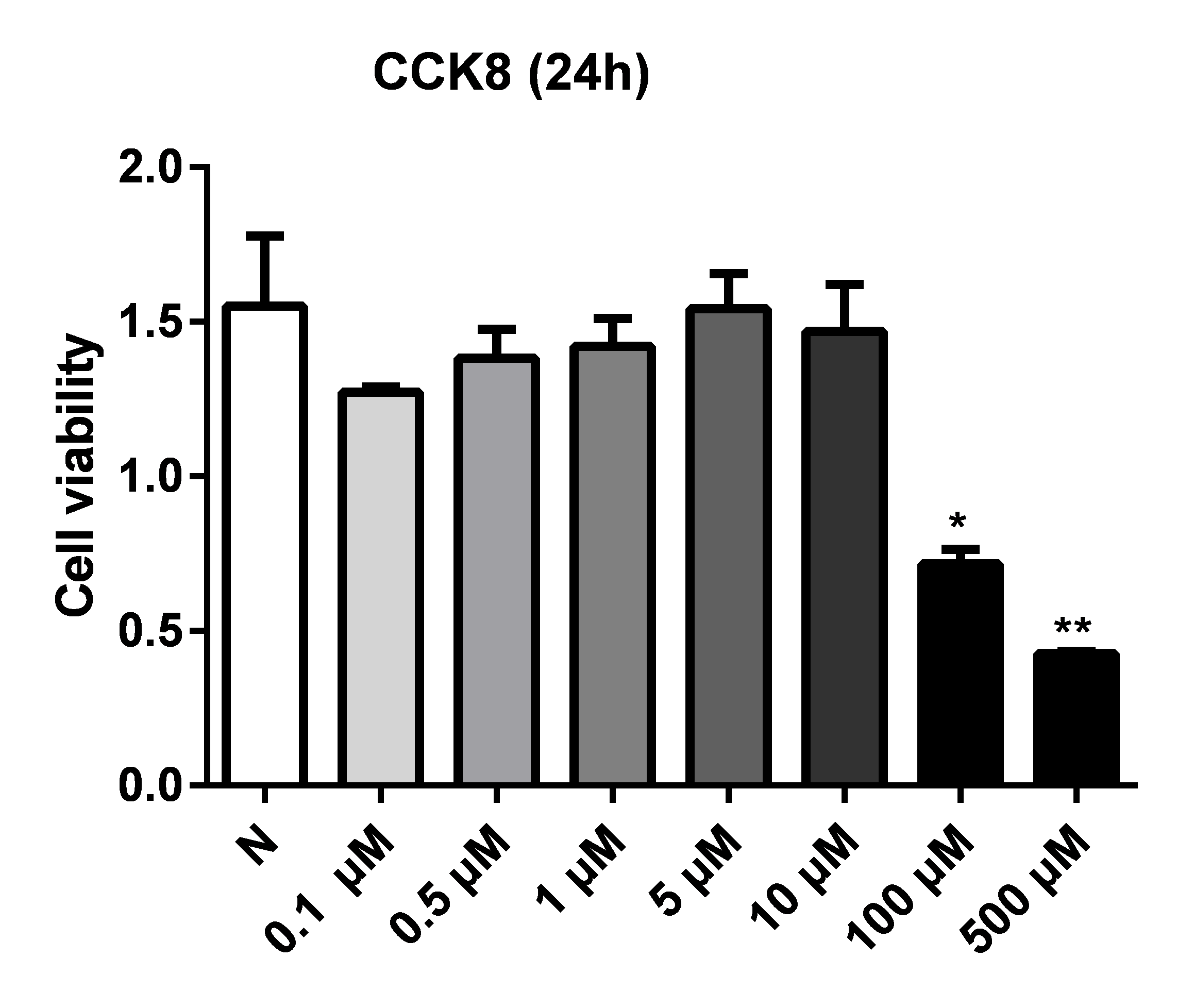


Murine ANA-1 macrophages were cultured in RPMI 1640 medium (1×10^5^ cells/ml, 1×10^4^ cells per well in 96-well plate) for 24 hours and then transferred to BA solution. After 48 hours incubation, the cell viability was determined by CCK8 method.
